# ATM/ATR-dependent Tyr15 phosphorylation of cyclin-dependent kinases in response to hydroxyurea in *Vicia faba* root meristem cells

**DOI:** 10.1007/s00709-013-0490-2

**Published:** 2013-03-07

**Authors:** Konrad Krajewski

**Affiliations:** Department of Cytophysiology, Faculty of Biology and Environmental Protection, University of Lodz, ul. Pomorska 141/143, 90-236 Lodz, Poland

**Keywords:** Cyclin-dependent kinase, Hydroxyurea, Tyrosine phosphorylation, Cell cycle checkpoint, *Vicia faba*

## Abstract

DNA damage or stalled replication forks activate cell cycle checkpoints. However, the regulation of metabolic pathways that are responsible for maintenance of genome integrity in plants is still largely unknown. Present research on *Vicia faba* root meristem cells indicates that inhibitory phosphorylation of cyclin-dependent kinases (Cdks) at Tyr15 plays a prominent role during blockage of cell cycle in response to genotoxic stress. Phosphorylation of P-loop in Cdks takes place in ATM/ATR-dependent manner. Although, Tyr15 phosphorylation upon hydroxyurea (HU) treatment was found in most cells classified to G1 and S phase, interestingly, the number of phoshpo-Tyr15-positive cells decreases in G2 phase. Presented data confirm much similarity in regulation of Cdks functions under genotoxic stress between plants and animals; however, they may also substantiate evolutionarily developed differences especially in regulation of G2/M transition between these two kingdoms.

## Introduction

Animals and plants show high homology of metabolic pathways that underlie cell cycle regulation. A major role in this process is played by key regulators, such as cyclin-dependent kinases (Cdks) and their partners, cyclins. However, in the course of evolution, some divergence appeared between these two kingdoms. In plants, Cdks have been classified into six distinct groups (CdkA to CdkF); however, some of them have no homologs in animals (Vandepoele et al. 2002). CdkA plays a role both in transition from G1 to S phase and from G2 to M phase (Burssens et al. 2000). Activity of plant-specific CdkB seems to be implicated in G2/M transition, since it accumulates preferentially in G2 phase. Moreover, two forms of CdkB were indentified in *Arabidopsis thaliana*, i.e., CdkB1 and CdkB2 (Porceddu et al. 2001; Francis 2011). CdkA and CdkB2 may interact with distinct cyclins involved in cell cycle regulation, e.g., cyclins A, B, or D (Kono et al. 2003). In metazoans, an appropriate level of Cdks is achieved before transition to the next phase of cell cycle owing to the fact that kinases are kept inactive by inhibitory phosphorylation of Thr14 and Tyr15 residues in the P-loop. Thus, activation of Cdk/cyclin complexes, next to phosphorylation of Thr161 in the T-loop, requires also Cdc25-dependent dephosphorylation of Thr14 and Tyr15 (Qu et al. 2003; Timofeev et al. 2010).

Regulation of Cdks activity also becomes prominent during cellular response to genotoxic stress. DNA damage triggers activation of cell cycle checkpoints established by ATM/ATR sensor kinases and their downstream factors Chk1/Chk2. In metazoans, the latter proteins mediate inhibitory phosphorylation of Cdc25 phosphatases (Abraham 2001; Vermeulen et al. 2003, Sancar et al. 2004). Hence, activation of the cell cycle checkpoints prevents dephosphorylation of the P-loop region, leading, finally, to cell cycle arrest.

In *Arabidopsis*, Tyr15 phosphorylation has been shown on CdkA;1, CdkD;2, and CdkD;3 during in vitro analysis (Shimotohno et al. 2006). Furthermore, inhibitory phosphorylation of CdkA;1 has been also revealed in hydroxyurea (HU)-treated plants (De Schutter et al. 2007). However, despite a confirmed role of Thr161 phosphorylation in T-loop of CdkA (Dissmeyer et al. 2007), the regulatory function of an inhibitory phosphorylation in the P-loop of this protein is questioned (Dissmeyer et al. 2009). Moreover, plant Cdc25 was found to act as arsenate reductase and its role in dephosphorylation of plant Cdks is not clear (Francis 2011; Spadafora et al. 2011).

The aim of this work is to determine whether Tyr15, a regulatory residue of Cdks, is phosphorylated in response to HU-induced DNA damage in *Vicia faba* root meristem cells, and if so, whether ATM/ATR-dependent checkpoint pathways participate in this modification. To check this, caffeine (CF), an inhibitor of plant ATM/ATR kinases (Smetana et al. 2012) was used. Since in animals, p38 kinase may modulate the functioning of Cdc25 phosphatases (Astuti et al. 2009; Thornton and Rincon 2009) and some data point to the activity of p38-like kinase in plants (Capone et al. 2004; Komis et al. 2004; Jiang et al. 2008), the effect of SB202190, p38 kinase inhibitor, on the level of Cdks phosphorylation under genotoxic stress was investigated. Another goal of this study was to determine whether there are any changes in the number of phospho-Tyr15-positive cells upon HU treatment in a course of the cell cycle.

If we assume that DNA damage triggers inhibitory phosphorylation of Cdks in plants, an important question arises about how further reduction of kinase phosphorylation is carried on in order to resume progression throughout the following stages of the cell cycle when genotoxic stress abates. Due to uncertain participation of Cdc25 phosphatases in dephosphorylation of plant Cdks, it was investigated whether continuation of the cell cycle after release from HU block results from the time-dependent proteasomal degradation of phosphorylated kinases and their replacement by newly synthesized, nonphosphorylated Cdks. To check this, HU-treated seedlings were shifted either to water or to a solution of proteasome inhibitor (MG132).

## Materials and method

### Material

*V. faba* subsp. *minor* var. *Nadwiślański* were sown on wet filter paper in Petri dishes and germinated for 3 days at room temperature in darkness. For experiments, seedlings with roots ranging from 1.5 to 2.0 cm in length were selected and incubated for 24 h in water (control), 2.5 mM HU, 80 μM SB202190, 5 mM CF, mixture of 2.5 mM HU and 80 μM SB202190, or mixture of 2.5 mM HU and 5 mM CF. To determine whether resumption of cell cycle after release from HU depends on proteasomal degradation of phosphorylated Cdks, HU-treated seedlings (24 h) were postincubated in water or 50 μM MG132 for 1, 2, 4, and 8 h.

### Chemical agents

Hydroxyurea, MG132, ATP, protease inhibitor cocktail, 1,4-diazabicyclo[2.2.2]octane (DABCO), 4',6-diamidino-2-phenylindole (DAPI), pectinase from *Aspergillus niger*, cellulase Onozuka R-10 from *Trichoderma viride*, Triton X-100, and Ponceau S solution were supplied by Sigma-Aldrich; pectolyase Y-23 by MP Biomedicals; and NuPAGE® Novex® 4–12 % bis–tris gel, polyvinylidene fluoride (PVDF) membrane (0.2-μm pore size), and Chromogenic Western Blot Immunodetection Kit by Invitrogen. P-PER Plant Protein Extraction Kit, Halt™ protease, and phosphatase inhibitor cocktail were obtained from Thermo Scientific. Other chemicals were obtained from POCH S.A.

### Western blotting

Proteins were extracted from apical root parts (3 mm) with the use of P-PER Plant Protein Extraction Kit supplemented with protease inhibitor cocktail. Extracts were fractionated on NuPAGE® Novex® 4–12 % bis–tris gel and then blotted onto PVDF membrane (0.2-μm pore size). Phosphorylation of Cdks at Tyr-15 was detected using monoclonal anti-phospho-cdc2 (Tyr15) antibodies diluted at 1:1,000 (Cell Signaling) and secondary goat anti-rabbit IgG antibody conjugated with alkaline phosphatase (Chromogenic Western Blot Immunodetection Kit). For total protein detection, PVDF membranes were stained with Ponceau S stain for 1 h.

### Immunodetection of Cdks phosphorylation at Tyr15

Apical root parts (1.5 mm) were fixed in phosphate-buffered saline (PBS)-buffered 4 % paraformaldehyde (4 °C; pH 7.4) for 45 min and then rinsed twice in PBS and transferred for 45 min to the citrate-buffered mixture (pH 5.0; 40 °C) containing 2.5 % pectinase from *A. niger*, 2.5 % cellulase Onozuka R-10 from *T. viride*, and 2.5 % pectolyase Y-23. Next, root tips were rinsed twice in cold PBS, squashed onto microscope slides (SuperFrost, Menzel-Gläser) in a drop of distilled water, and placed on dry ice. After 10 min, cover slips were removed and the slides were washed with PBS, distilled water, and air dried. Macerated cells were permeabilized with 0.5 % Triton X-100 for 15 min, preincubated in the blocking buffer (5 % BSA, 0.3 % Triton X-100, PBS) and then incubated overnight (4 °C) with primary monoclonal anti-phospho-cdc2 (Tyr15) antibodies (1:25, Cell Signaling) dissolved in the antibody dilution buffer (1 % BSA, 0.3 % Triton X-100, PBS). After that, slides were washed in PBS and incubated at room temperature for 90 min with secondary fluorescein isothiocyanate (FITC)-conjugated anti-rabbit IgG (whole molecule; 1:350, Sigma-Aldrich) dissolved in the antibody dilution buffer (1 % BSA, 0.3 % Triton X-100, PBS). Nuclear DNA was stained with DAPI (15 μM, 15 min) and then the slides were washed in PBS. The specimens mounted in PBS/glycerol mixture (9:1) containing 2.5 % DABCO were photographed using an Eclipse E600W microscope (Nikon). DM 505 filter (excitation wavelength 465–495 nm) and DM 400 filter (excitation wavelength 340–380 nm) were used for FITC and DAPI, respectively.

### Measurements and statistical analysis

Mean immunolabeling indices (including the values calculated for the whole cell populations and for successive phases of the cell cycle) and median values of immunofluorescence intensity were evaluated based on the analyses of the four most representative roots (selected out of six) for each experimental series. Total labeling index expresses the ratio of cells with specific fluorescence to all cells in the examined population (1,500–2,000 cells per root). The labeling index for each phase of the cell cycle indicates the ratio of labeled cells to all cells in a particular stage of interphase (analyzed jointly for all roots, 300–1,000 cells). For semiquantitative measurements, immunofluorescence micrographs were converted to grayscale and analyzed using the computer-aided Cytophotometer v1.2 (Forel, Lodz, Poland). Median values of phospho-Tyr15 immunofluorescence intensity (analyzed jointly for all roots) were estimated based on 300 to 1,000 individual measurements, depending on the phase of the cell cycle. Fluorescence intensity obtained from a single nucleus expresses the mean pixel value spanning the range from 0 to 255, multiplied by the nuclear profile surface area. In each experiment, cell cycle positions were assigned according to different intensities of DAPI-DNA fluorescence. Statistical analysis was performed using STATISTICA 10 PL software.

## Results and discussion

### Genotoxic stress induces Cdks phosphorylation at Tyr15 in ATM/ATR-dependent manner

**Fig. 1:**
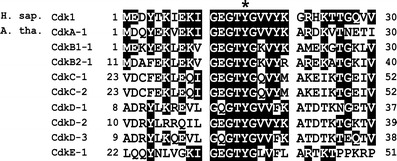
Comparison of amino acid sequences of Cdk1 from *Homo sapiens* and different types Cdks from *Arabidopsis thaliana* (basing on UniProt database). *Asterisk* represents conservative phosphorylated Tyr15 residue. *White-on-black letters* indicate similarity of amino acid residues in vicinity of Tyr15. *Numbers* indicate the position of amino acid residues in the sequences. (Shimotohno et al. 2006)

**Fig. 2:**
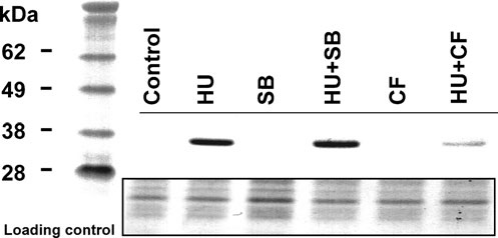
Immumoblotting analysis of phosphorylated Cdks at Tyr15 in whole-cell extracts. Plants were incubated in water (control) or incubated for 24 h in: 2.5 mM hydroxyurea (HU), 80 μM SB20190 (SB), mixture of 2.5 mM hydroxyurea and 80 μM SB20190 (HU + SB), and 5 mM caffeine (CF) and mixture of 2.5 mM hydroxyurea and 5 mM caffeine (HU + CF). Loading control represents level of proteins with molecular mass in the range of 14–28 kDa, detected with Ponceau S stain

Immunocytochemical and biochemical analyses were performed with the use of monoclonal antibodies raised against human Cdk1 phosphorylated at Tyr15. Due to considerable similarities in the conservative region of the P-loop observed among plants and animals, it is likely that the applied antibodies detect Tyr15 phosphorylation of only one or several Cdks in *V. faba* (Fig. 1). Western blot analyses revealed high level of Tyr15 phosphorylation in extracts from seedlings treated both with HU and the mixture of HU and SB202190. More importantly, plants incubated jointly with HU and CF show considerable reduction of inhibitory phosphorylation. No immunosignals were found in extracts from control, SB202190-, and CF-treated seedlings (Fig. 2).

**Fig. 3:**
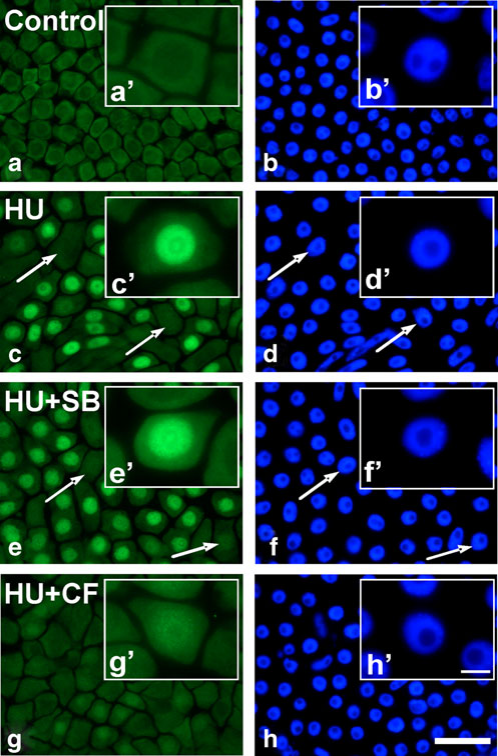
Immunocytochemical detection of phosphorylated Cdks at Tyr15. **a**, **b** Incubation in H_2_0. **c**, **d** 24-h incubation with 2.5 mM HU. **e**, **f** 24-h incubation with mixture of 2.5 mM HU and 80 μM SB20190. **g**, **h** 24-h incubation with mixture of 2.5 mM HU and 5 mM CF. **a**, **c**, **e**, **g** Immunolabeling. **b**, **d**, **f**, **h** DAPI staining. *Arrows* point to cells with unlabeled nuclei. **a**–**h** bar 50 μm, **a**′–**h**′ bar 10 μm

**Fig. 4:**
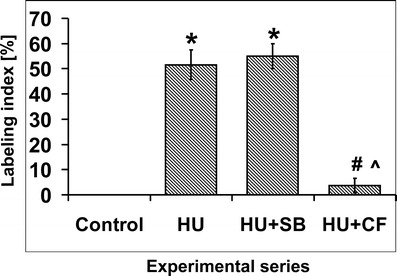
Total labeling indices (%) estimated for *V. faba* root meristem cells stained with anti-phospho-Cdk1 (Tyr15) antibodies. Plants were incubated in: water [control], 2.5 mM HU for 24 h [HU], mixture of 2.5 mM HU and 80 μM SB20190 for 24 h [HU + SB], and the mixture of 2.5 mM HU and 5 mM CF for 24 h [HU + CF]. *Error bars* represent standard deviation. Statistical significance (Mann–Whitney test, *p* < 0.05): *asterisk* control/HU and control/HU + SB; *number sign* HU/HU + CF and HU + SB/HU + CF; *circumflex accent* control/HU + CF

Fluorescence microscopy observations indicated that the immunofluorescence signals were limited to nuclei. Interestingly, not all cells displayed inhibitory modification of the P-loop in response to HU treatment (Fig. 3). Analyses of labeling indices confirm the results obtained by Western blotting. It has been shown that cells from control plants were free of labeling, in contrast to other experimental series. Plants incubated both in HU and in the mixture of HU and SB revealed high labeling indices, 51 % and 55 % respectively. Notably, administration of CF upon HU treatment brought about reduction of labeling index to 4 % (Fig. 4).

Presented data showing Tyr15 phosphorylation as a result of response of *V. faba* seedlings to HU are consistent with earlier research performed on plants (De Schutter et al. 2007) as well as on animals and yeasts (Rhind and Russell 1998; Zarzov et al. 2002; Zhang et al. 2008). In turn, reduction of HU-induced inhibitory phosphorylation by CF point to the role of ATM/ATR-dependent checkpoints in the regulation of plant Cdks activity upon DNA damage. A similar effect of CF was previously observed in animals (Wang et al. 1999; Darbon et al. 2000). The decrease of HU-induced Tyr15 observed following chemical inhibition of ATM/ATR kinases conforms to previous data obtained with the use of *Arabidopsis atm-1* and *atr-2* mutants (De Schutter et al. 2007). On the other hand, Dissmeyer et al. (2009) point out that DNA damage cell cycle checkpoints function independently of Thr-14 and Tyr-15 phosphorylation of plant CdkA.

In contrast to the effect driven by CF, SB202190, an inhibitor of animal p38 kinase, did not decrease the extent of HU-induced phosphorylation of Tyr15. This seems to support an earlier hypothesis that p38 kinase inhibitor does not overcome the DNA damage-induced cell cycle checkpoints in plants. Thus, previously observed increase in the number of aberrant mitoses in plants treated jointly with HU and SB202190 may result from impact that SB202190 exerts on the expression of genes responsible for DNA damage repair (cf. Krajewski and Maszewski 2012).

In contrast to the data obtained by Dissmeyer et al. (2009), current results allow to conclude that inhibitory phosphorylation of the P-loop takes part in HU-mediated cell cycle arrest rather than plays an important role during progression throughout the cell cycle. CF-induced effects clearly indicate that Tyr15 phosphorylation upon HU treatment takes place in ATM/ATR-dependent manner and strongly substantiate the results presented by De Schutter et al. (2007). In contrast, SB202190 has no effect on the level of HU-induced Tyr15 phosphorylation. This may suggest that p38-like proteins do not participate in the regulation of cell cycle checkpoints in plants.

### HU-mediated Tyr15 phosphorylation appears mostly in the G1 and S phase cells

**Fig. 5:**
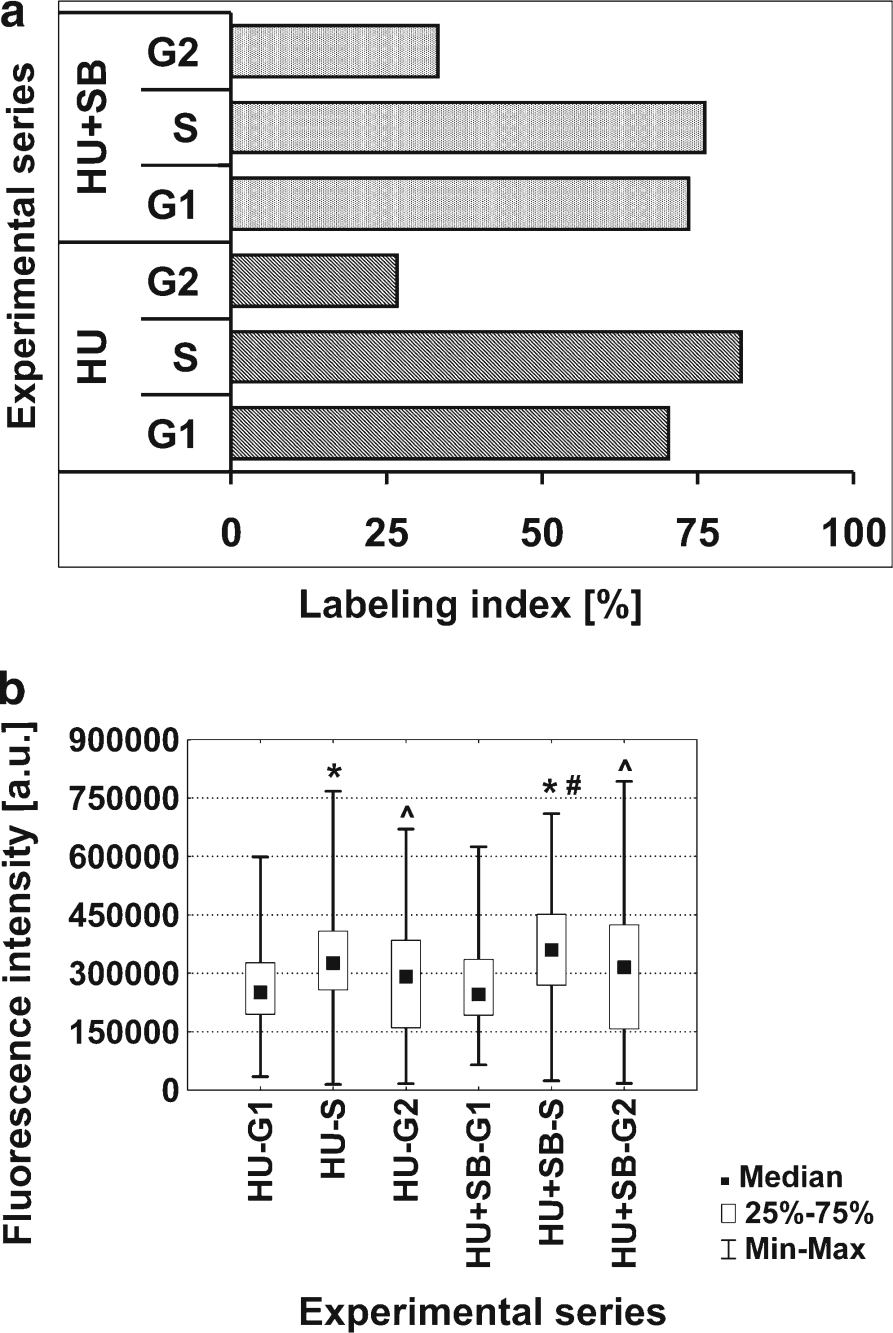
Labeling indices in the individual phases of cell cycle (**a**) and median fluorescence intensity [arbitrary units, a.u.] (**b**) estimated for *V. faba* root meristem cells stained with anti-phospho-Cdk1 (Tyr15) antibodies. Successive phases of the cell cycle in seedlings incubated with 2.5 mM HU, denoted as HU-G1, HU-S, and HU-G2, and in seedlings incubated with the mixture of 2.5 mM HU and 80 μM SB, denoted as HU + SB-G1, HU + SB20190-S, and HU + SB-G2. Statistical significance in **b** (Kruskal–Wallis test, *p* < 0.01): *asterisk* HU-G1/HU-S, HU + SB-G1/HU + SB-S; *number sign* HU-S/HU + SB-S; *circumflex accent* HU-S/HU-G2, HU + SB-S/HU + SB-G2

Apart from total immunolabeling indices, the percentage of labeled nuclei in each phase of cell cycle was also estimated (Fig. 5a). Obtained results show that HU treatment has triggered Tyr15 phosphorylation in the vast majority of cells classified to G1 and S phases. In this case, labeling indices reached 70 % and 82 %, respectively. Interestingly, the number of labeled cells decreased in G2 phase and only 27 % of those cells displayed characteristic immunofluorescence upon HU treatment. Seedlings incubated in the mixture of HU and SB202190 show comparable values of labeling indices to those evaluated for HU-treated plants. Similarly, both G1 and S phase cells displayed high level of labeling indices, 73 % and 76 %, respectively. In G2 phase, the number of phospho-Tyr15-positive cells reached 33 % only.

Semiquantitative measurements evaluated for each phase of cell cycle indicate that the intensity of immunofluorescence observed in S phase cells is slightly higher than that registered for G1 and G2 phase cells upon HU treatment. Similarly, seedlings incubated jointly in the mixture of HU and SB202190 displayed the highest median fluorescence intensity in S phase cells. Interestingly, a statistically significant difference between two experimental series was observed in S phase only (Fig. 5b). Due to low total labeling index evaluated for seedlings treated jointly with HU and CF, this experimental series was omitted during the analyses of percentage of labeled cells in successive phases of the cell cycle and quantitative measurements of immunofluorescence intensity.

The high number of phospho-Tyr15-positive cells in S phase seems to confirm, indirectly, involvement of wee1 kinase in the course of S phase and during the intra-S phase checkpoint (Mendes et al. 2005; Cools et al. 2011). However, high labeling index in G1 phase indicates that Tyr15 may regulate Cdk activity upon genotoxic stress not only in S phase but also in G1 phase. Furthermore, reduction in number of immunolabeled G2 phase cells following HU treatment seems to support a previous hypothesis that during G2/M transition in plants, the key role is played by B- and A-type Cdks, with the activity of the latter regulated by Cdks inhibitors (CKI) (Menges et al. 2005; Francis 2011; Cools et al. 2011). It cannot be excluded that upon HU treatment, Cdks are regulated by Tyr15 phosphorylation in both G1 and S phases as well as in early stages of G2 phase. During late G2 phase and G2/M transition, cells may be protected from prematurely induced mitosis by CKI, without involving Tyr15 phosphorylation.

### Decrease in Tyr15 phosphorylation after release from HU is regulated in a time-dependent manner and is not connected with Cdks degradation by proteasome

Since the role of Cdc25 phosphatases in dephosphorylation of plant Cdks is not clear, it was investigated whether continuation of the cell cycle after release from HU is a result of proteasomal degradation of phosphorylated kinases and their replacement with a pool of newly synthesized, nonphosphorylated Cdks. To check this hypothesis, HU-treated seedlings were postincubated either in water or in solution of proteasome inhibitor (MG132) for 1, 2, 4, and 8 h.

**Fig. 6:**
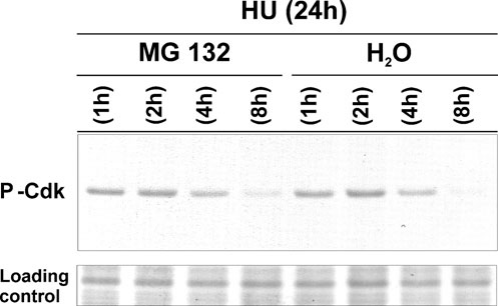
Immumoblotting analysis of phosphorylated Cdks at Tyr15 in whole-cell extracts. Plants were incubated in 2.5 mM HU for 24 h and postincubated in 50 μM MG132 or water for 1, 2, 4, and 8 h, respectively. Loading control represents level of proteins with molecular mass in the range of 14–28 kDa, detected with Ponceau S stain

The obtained results revealed the time-dependent decrease of Tyr15 phosphorylation during successive hours of postincubation in water (Fig. 5). The observed effect is consistent with previous data (Corellou et al. 2005). Moreover, total reduction of Tyr15 phosphorylation at 8 h may also explain why, in *V. faba* root meristem cells, the burst of mitotic cells was observed just 8 h after release from HU block (cf. Rybaczek et al. 2007). The presence of MG132 triggered a similar effect, and only at 8 h after release, Cdks dephosphorylation was found slightly limited (Fig. 6). Therefore, the obtained results seem to indicate that after HU release, cell cycle resumes independently of the proteasomal degradation of Cdks. It is possible that Cdk dephosphorylation takes place in a phosphatase-dependent manner; however, other mechanisms may also reduce the pool of phosphorylated Cdks.

## Conclusion

Summing up, the present data seem to imply that plants and animals share similar mechanisms underlying HU-induced cell cycle arrest. Phosphorylation of Cdks in the P-loop during HU treatment and reduction of this modification upon treatment with the mixture of HU and CF indicate that inhibitory phosphorylation of Cdks at Tyr15 plays a crucial role during ATM/ATR-dependent response to genotoxic stress in plants. Accurate metabolic pathway linking ATM/ATR kinases and Cdks remains unknown. Due to the lack of Chk1 and Chk2 kinases in plants (Francis 2011) and considering the known enhancement of *Wee1* gene expression under genotoxic stress (De Schutter et al. 2007), it seems very probable that in presence of DNA damage, ATM/ATR kinases directly activate Wee1 kinase, which in turn, executes Tyr15 phosphorylation of plant Cdks. Interestingly, opposite to G1 and S phase cells, only a small number of G2 phase cells displayed Tyr15 phosphorylation in response to HU treatment. This fact may support an earlier hypothesis that plants regulate G2/M transition differently from animals. It may be that Cdks activity in G2 phase is mostly regulated by CKI, with only minor involvement of Tyr15 phosphorylation (cf. Francis 2011; Cools et al. 2011).
